# Mineralogical, Textural and Physical Characterisation to Determine Deterioration Susceptibility of Irulegi Castle Lime Mortars (Navarre, Spain)

**DOI:** 10.3390/ma12040584

**Published:** 2019-02-15

**Authors:** Graciela Ponce-Antón, Anna Arizzi, Maria Cruz Zuluaga, Giuseppe Cultrone, Luis Angel Ortega, Juantxo Agirre Mauleon

**Affiliations:** 1 Department of Mineralogy and Petrology, Faculty of Science and Technology, University of the Basque Country-UPV/EHU, Sarriena s/n, 48940 Leioa, Bizkaia, Spain; mcruz.zuluaga@ehu.eus (M.C.Z.); luis.ortega@ehu.eus (L.A.O.); 2 Department of Mineralogy and Petrology, Faculty of Sciences, University of Granada, Avda. Fuentenueva s/n, 18002 Granada, Spain; arizzina@ugr.es (A.A.); cultrone@ugr.es (G.C.); 3 Aranzadi Society of Sciences, Zorroagagaina 11, 20014 Donostia-San Sebastián, Gipuzkoa, Spain; zuzendaritza@aranzadi.eus

**Keywords:** lime mortar, mineralogy, texture, durability, deterioration, hydric behaviour, pore system

## Abstract

Archaeological lime mortars from the Tower Keep and West perimeter wall of Irulegi Castle (Navarre, Spain) were analysed to determine susceptibility to deterioration. Chemical, mineralogical, textural and physical characterisation was performed by different tests and multianalysis techniques in order to determine the intrinsic features of the original historical mortars at the castle. Samples from the Tower Keep are more prone to deteriorate compared with the West perimeter wall due to high water absorption capacity and high porosity. A high degree of pore interconnection, high desorption index and the presence of high pore volume in the 0.01 to 1 µm size range affect the mortar durability since pores retain water longer inside the mortar. Local environment conditions with persistent annual rainfall, high humidity and temperature variations contribute to the decay process of the original mortar. Characterisation of historical mortars not only allows better understanding of susceptibility to deterioration but also helps the design of compatible and durable repair mortar for future interventions on historical heritage. Compatibility of new materials with the historical mortar will be ensured by studying mortar characteristics and properties.

## 1. Introduction

The preservation of built heritage requires suitable materials and techniques to enable effective restoration interventions [1]. The characterisation of original historical mortars is an important step before carrying out any repair interventions since the characteristics of the new mortar must be as similar as possible to those of the ancient mortar [2]. Differences in the material properties lead to a lack of compatibility between the new and original mortar reducing their durability [3]. Several authors have carried out chemical, mineralogical and physical analyses prior to the formulation of repair mortars [4,5,6,7]. Comparative studies using different limes and additives also have been carried out [8,9,10]. In addition, the material is exposed to different environmental conditions to determine how they affect the mortar properties and which factors are involved in their decay [11,12,13,14,15]. Studies of the repair material properties are performed in order to select the most suitable mortar mixture for restoration work [16].

Environmental factors condition material deterioration processes [11,17]. Water, in liquid or vapour form (e.g., as humidity), favours the irreversible phenomenon of decay, giving place to different physical, chemical and biological deterioration processes [18,19]. Chemical degradation of mortars takes place mainly due to hydrolysis, hydration or oxidation processes. Volume increase within the pores, by such processes as crystallization of water into ice or swelling of some clay minerals, leads to physical degradation of the material [20]. Furthermore, water can incorporate dissolved salts into the material that may crystallize after water evaporation, as well as gaseous species such as CO_2_ that can dissolve the calcareous materials under specific conditions [21].

Durability of materials does not only depend on the environmental factors but also on their intrinsic mineralogical and textural features [22,23]. The pore system plays an important role in mortar durability since weathering processes often depend on the circulation of water inside the pores, accelerating the physical, chemical and biological deterioration [24]. Studies on hydric behaviour have been carried out to understand water deterioration mechanisms in building materials since the parameters associated with fluid uptake and transport inside the pores directly influence material deterioration [3,5]. Water circulation through the material is also conditioned by the presence of anisotropies and the interconnection degree between the pores [17,25].

Not only compatibility but also authenticity of the restoration material with the original mortar is one of the main goals in heritage conservation. Achieving aesthetic features in terms of visual appearance (e.g., texture and colour) is another important requirement in the restoration process [2,26]. Colour is a sensorial perception between the object, the lighting and the observer, so visual variations in colour between restored and original materials is an issue of interest in restoration [27,28]. In addition to chemical, mineralogical, physical and aesthetic characterisation, the workability is another important feature to consider in mortar formulation [26].

Irulegi Castle (Navarre, northern Spain) was built in a defensible site. Geographically the castle is located in a mid-latitude climate zone with a suboceanic west coastal maritime climate [29]. Regional climate is characterised by a moderately warm climate with cool summers and abundant rainfall well distributed throughout the year, although with two dry months.

The aim of this study is to assess the hydric behaviours of lime-based archaeological mortars from Irulegi Castle in order to establish their susceptibility to deterioration. Taking into account the climatic conditions to which historical mortars are exposed, knowledge of chemical, mineralogical and physical properties will allow the formulation of an adequate repair mortar to ensure the compatibility and authenticity of the restoration material with the original mortar.

## 2. Archaeological Background

**Figure 1:**
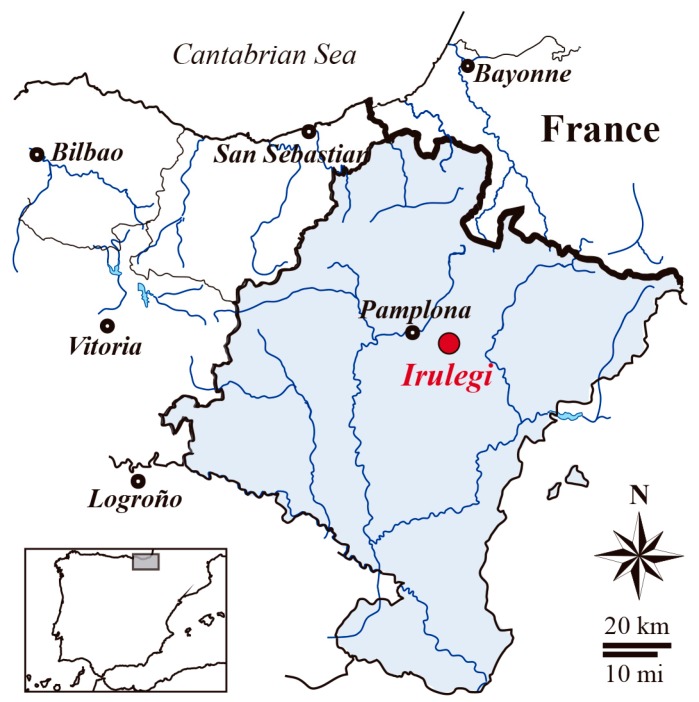
Geographic location of Irulegi Castle (Navarre, Spain).

The medieval archaeological site of Irulegi Castle is on the eastern border of the Pamplona Basin (Figure 1). It is a rock castle on Irulegi Mountain in the east of Aranguren mountain range (Navarre, Spain). The historical strategic emplacement of the castle allowed visual control of the Navarre kingdom capital and the routes to the pass over the Pyrenees along the Izagaondoa valley.

**Figure 2:**
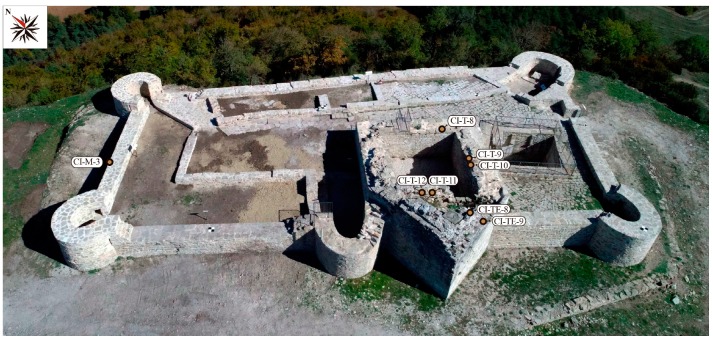
Location of studied samples in the Tower Keep and West perimeter wall at Irulegi Castle.

The walled archaeological site is characterised by a rectangular floor 39 m × 15 m in size with an area of approximately 460 m^2^. Within the castle structures, mortars from the Tower Keep and the West perimeter wall were studied. The Tower Keep presents pseudoisodom bonding with high quality ashlars. The absence of compositional differences in the tower ashlars indicates that all the structure was built in the same construction period [30]. Nevertheless important renovations in the defensive elements of the castle were undertaken as a result of the constructive techniques developed over time. The remodelling of the Tower Keep resulted in the dismantling of the south outer wall and the original rectangular floor was transformed into the current polygonal floor with a vertex-shaped structure. This remodelling increased the floor area from 90 m^2^ to 105 m^2^. The west perimeter wall shows lower-quality irregular bonding and has average dimensions of 1.25 m width and 11.35 m length (Figure 2). A continuous settlement sequence from the Late Bronze Age to the Late Middle Ages has been recognised in the archaeological site. However, the earliest fortification building is unknown since no documentary data about the first building period of the castle exist. The oldest mentions of Irulegi Castle are dated in the second half of the 12th century and beginning of the 13th century. The castle was demolished at the end of the 15th century and currently only ruins remain standing [30,31].

## 3. Materials and Methods

### 3.1. Materials

Eight archaeological mortar samples, seven from the Tower Keep and one from the West perimeter wall at Irulegi Castle (Navarre, Spain) were collected and analysed (Table 1 and Figure 2). While the Tower Keep structure is still unconsolidated, the West perimeter wall was consolidated in 2017 but the sample corresponding to this wall was collected before the consolidation of the castle ruins. Considering the studied mortars correspond to archaeological materials, sample selection was determined on the basis of the minimum volume required to perform the capillary tests.

### 3.2. Methods

Different analytical techniques and tests were performed to determine the mineralogy, chemistry, texture and physical properties of the collected archaeological mortars.

#### 3.2.1. Mineralogical, Chemical and Petrographic Characterisation

The mineralogical composition of samples was determined by means of X-ray diffraction (XRD) using a Philips X’Pert diffractometer (Leioa, Spain) equipped with a monocromatic Cu-k_α1_ X-radiation operating at 40 kV and 20 mA. The data collection on the powder sample was performed by a continuous scan in the range from 5 to 70° 2θ, at an acquisition rate of 0.02° per second. Mineral phase identifications were performed with X’Pert HighScore Plus 3.0 software by PANalytical (Leioa, Spain).

Chemical composition of major elements in bulk mortar was established by means of X-ray Fluorescence (XRF) in powder sample. Measurements were taken by Wavelength Dispersive X-ray Fluorescence (WDXRF) using a PANalytical Axios Advanced PW4400 XRF spectrometer (4 kW Rh anode SST-mAX X-ray tube, Leioa, Spain). Fused beads were obtained after heating a sample and lithium borate flux (Spectromelt A12, Merck, Leioa, Spain) mixture in approximate 20:1 proportions at ~1200 °C for 3 min in Pt/Au crucibles using a PANalytical Perl’X3 fusion machine. Detection lower limits for major elements are in the range of 0.01 wt %. The loss on ignition (LOI) has been calculated after heating a powder sample of bulk mortar at 1050 °C for one hour.

The mortar texture and nature of components were determined in polished thin sections using a Nikon Eclipse LV100POL microscope (Leioa, Spain) equipped with DS F-I1 digital camera and a DS L-2 control unit.

#### 3.2.2. Characterisation of Pore System and Hydric Behaviour

Mercury intrusion porosimetry (MIP) was used to determine the pore size distribution and the open porosity (P_MIP_) by a Poremaster-60 GT (Quantachrome Instruments, Alicante, Spain), with a maximum injection pressure of 414 MPa, measuring the pore diameter range from approximately 0.003 to 360 µm. Mortar sample fragments about 1 cm^3^ were oven-dried for 24 h at 60 °C before the analysis.

To obtain a complete vision of the pore system, hydric tests (HT) were carried out in samples of 3 cm^3^ in size; previously oven-dried at 80 °C for 24 h. Measurements were taken under controlled thermo-hygrometric conditions at 25 °C and 50% relative humidity. The test measurements were performed on no more than two or three samples per mortar type, since samples are archaeological materials.

The free (A_b_) and forced (A_f_, under vacuum) water absorption values and absorption coefficient (C_a_) were determined following the UNE-EN 13755 [32] standard. The degree of interconnection between the pores (A_x_) [33] and the saturation coefficient (S) [34] were also determined. These hydric parameters were calculated as follows

(1) $$A_{b}=\frac{M_{L}-M_{0}}{M_{0}}\cdot 100$$

where M_0_ is the mass of the dried sample and M_L_ is the mass of the sample saturated under water at atmospheric pressure (until constant mass is reached):

(2) $$A_{f}=\frac{M_{s}-M_{0}}{M_{0}}\cdot 100$$

(3) $$A_{x}=\frac{A_{f}-A_{b}}{A_{f}}\cdot 100$$

(4) $$C_{a}=\frac{A_{b}}{\sqrt{t}}$$

where M_S_ is the mass of the sample saturated with water under vacuum. The absorption coefficient (C_a_) is determined as the slope of the curve representing the weight increase as a function of the square root of time 4 min after the beginning of the test:

(5) $$S=\frac{M_{48h}-M_{0}}{M_{S}-M_{0}}\cdot 100,$$

where M_48h_ is the mass of the sample after 48 h immersion in water at atmospheric pressure.

Drying index (D_i_) is defined as the definite integral of the drying curve from the beginning (t_0_) to the end (t_f_) times of the test in which M_t_ represents a decreasing water weight content starting from the saturation values (under vacuum) as a function of time. The D_i_ was measured according to the NORMAL 29/88 [35]:

(6) $$D_{i}=\frac{\int_{t_{0}}^{t_{f}}f\;(M_{t})\mathrm{dt}}{M_{S}\cdot t_{f}}$$

The capillarity coefficient (C_c_) and the capillarity height (H_c_) of samples were calculated according to the UNE-EN 1925 [36] standard:

(7) $$C_{c}=\frac{M_{t}-M_{0}}{A\cdot \sqrt{t}},$$

where M_t_ is the amount of water absorbed at time t and A is the surface of the sample in contact with the water:

(8) $$H_{c}=\frac{h}{\sqrt{t}}$$

where h is the height of water rise by capillarity at time t.

Finally, UNE-EN 1936 [37] standard was used to determine the open porosity (P_HT_) and skeletal (ρ_Hsk_) and bulk (ρ_Hb_) densities as follows

(9) $$P_{\mathrm{HT}}=\frac{M_{s}-M_{0}}{M_{s}-M_{H}}\cdot 100$$

(10) $$\rho_{\mathrm{Hsk}}=\frac{M_{0}}{M_{0}-M_{H}}$$

(11) $$\rho_{\mathrm{Hb}}=\frac{M_{0}}{M_{s}-M_{H}},$$

where M_H_ is the mass of the sample saturated with water under vacuum and weighted in water.

#### 3.2.3. Nondestructive Tests

In order to assess mortar compactness, ultrasonic wave propagation was measured using a Control ultrasonic pulse velocity tester model 58-E4800 with a couple of nonpolarised piezoelectric transducers of 54 MHz (Granada, Spain). A viscoelastic couplant gel was applied to ensure a good contact between the transducers and the sample (Transonic gel). The propagation velocity of ultrasonic pulses (V_p_) was measured on samples of 3 cm^3^ in size (previously oven-dried at 80 °C for 24 h) according to the ASTM D 2845-05 [38] standard under controlled thermo-hygrometric conditions at 25 °C and relative humidity of 50%. V_p_ values were useful to determine the structural anisotropy (ΔM) [39] as follows:

(12) $$\Delta M=\left(1-\frac{{2V}_{\mathrm{pmin}}}{V_{\mathrm{pmax}}+V_{\mathrm{pmean}}}\right)\cdot 100,$$

where V_pmax_ is the mean maximum velocity, V_pmin_ is the mean minimum velocity and V_pmean_ is the mean intermediate velocity in each of the three orthogonal directions.

The colour characterisation of samples was carried out by spectrophotometry according to the CIELab system following the UNE-EN 15886 [40] standard. The lightness (L*) and chromatic (a* and b*) parameters were determined by means of a portable Konica-Minolta CM-700d spectrophotometer (8 mm diameter; D65 illuminant; 10° view angle; SCI/SCE mode; 400–700 nm light radiation range, Granada, Spain). Colour was measured in five points of each mortar sample and mean values were calculated.

## 4. Results and Discussion

### 4.1. Mineralogical, Chemical and Petrographic Studies

The mineralogy obtained by XRD shows calcite and quartz to be the most abundant mineral phases in all mortars, although in different relative amounts. Sample CI-T-9 displays the highest quartz content. Feldspars and phyllosilicates are present in all samples but in minor amounts. Only Samples CI-T-9 and CI-TE-9 contain iron oxides (hematite and goethite) corresponding to the rock fragments used as aggregates (Table 2).

Table 3 shows the quantitative chemical results of major elements determined by X-ray fluorescence. The chemical composition is in accordance with the identified mineral phases. High values of Si in all samples are related to such silicates as quartz, phyllosilicates and feldspars. In fact, sample CI-T-9 shows the highest SiO_2_ value (~41 wt %) corresponding to a quartz-enriched sample. On the contrary sample CI-M-3 has the lowest SiO_2_ value (~32 wt %). The high Ca content is mainly due to the presence of calcite, as well as the LOI value. CI-M-3 sample shows the highest CaO (~32 wt %) and LOI values (~29 wt %), indicating the major content of calcite in this sample. On the contrary, samples CI-T-9, CI-T-10 and CI-T-11 have the lowest Ca values (~26 wt %), indicating low carbonate content. Samples CI-M-3, CI-T-9, CI-T-10 and CI-T-11 display the highest Al, K, Mg and Ti values related to the relatively high phyllosilicate contents. Iron content is related to the presence of iron oxides/hydroxides (i.e., hematite and goethite) and phyllosilicates and the highest values correspond to samples with high Al, K, Mg and Ti values (i.e., samples CI-M-3, CI-T-9, CI-T-10 and CI-T-11).

**Figure 3:**
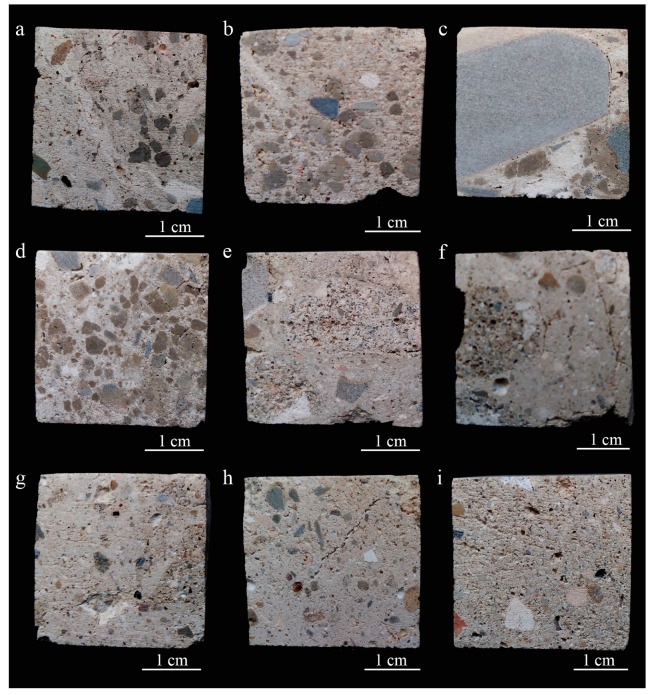
Images of archaeological mortar hand samples showing the heterogeneous texture. (**a**) Sample CI-T-8; (**b**) sample CI-T-10; (**c**) sample CI-T-9, mortar with a big size sandstone aggregate showing porosity at the grain edge; (**d**) sample CI-T-9, mortar with abundant presence of rock fragments; (**e**) sample CI-T-11, (**f**) Sample CI-T-12 and (**g**) sample CI-TE-9, mortars with reused mortar fragments; (**h**) sample CI-TE-8, cracked mortar; and (**i**) sample CI-M-3, showing the most homogeneous texture.

Textural and mineralogical differences of mortars imply variations in the hydric characteristics since the pore system could vary [41]. Macroscopically all samples show a heterogeneous texture both in aggregate size and nature (Figure 3a,b). Nevertheless, some samples contain aggregates that are bigger in size (Figure 3c) and in large amounts (Figure 3d) particularly in the case of Sample CI-T-9. Reused mortar fragments more than 1 cm in size (Figure 3e–g) and cracks can also be seen with the naked eye (Figure 3h). Within the heterogeneous texture of mortar samples, CI-M-3 has fewer aggregates in a more homogeneous texture compared with the other samples (Figure 3i). Porosity at the edge of larger aggregates can be observed (Figure 3c).

**Figure 4:**
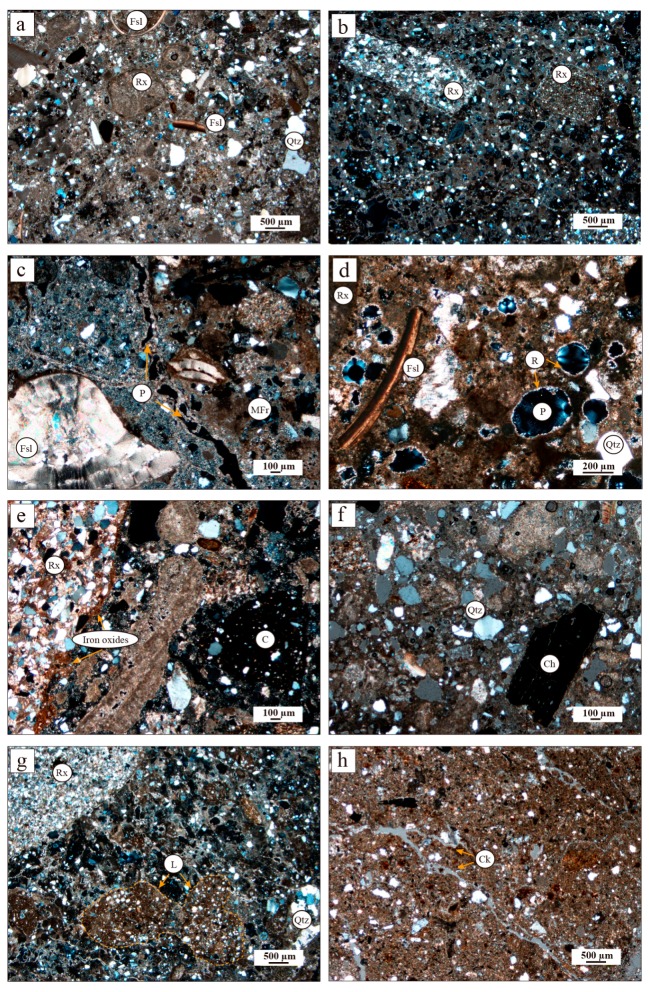
Photomicrographs show the most representative textural features of historic lime mortars from Irulegi Castle. (**a**) Mortar with quartz grains and rock and fossil fragments, (**b**) mortar with angular fragments of sandstone and (**c**) mortar with fossil (left) and reused mortar (right) fragments. Porosity between the mortar fragment and the matrix can be observed, (**d**) mortar with pores that show recrystallizations inside, (**e**) mortar with rounded ceramic fragment, (**f**) mortar with charcoal fragment, (**g**) mortar with quartz-bearing lumps and (**h**) mortar matrix with microcracks. C: ceramic, Ch: charcoal, Ck: crack, Fsl: fossil, L: lump, MFr: Mortar fragment, P: pore, Qtz: quartz, R: recrystallization, Rx: rock fragments.

The petrographic study improves the observed macroscopic features. Microscopically all the samples also exhibit heterogeneous texture (Figure 4). The matrix is formed by micritic calcite. Aggregates consist of heterometric and angular to subangular detrital quartz, sandstone, marl and calcarenite rock fragments (Figure 4a,b). A great variety of bioclast fragments (echinoderm plates, foraminifers and molluscs) are present due to the crushing of the rocks used as aggregate (Figure 4c,d). The quartz grains range from 0.05 µm to 0.25 µm in size. The smaller rock fragments are subangular while the largest are rounded (Figure 4b,e). Some sandstone fragments contain iron oxides (Figure 4e). Rounded ceramic fragments < 1 mm in size and charcoal fragments can also be seen dispersed in the matrix (Figure 4e,f). In samples CI-T-11, CI-T-12 and CI-TE-9, reused mortar fragments of <2 cm in grain size have also been observed (Figure 4c). Heterometric quartz-bearing lime lumps can be observed within the binder matrix (Figure 4g). The pores in the matrix are irregularly shaped and <200 µm in size and some show recrystallizations of secondary calcite inside (Figure 4d). Porosity at the edge of some aggregates can also be observed microscopically (Figure 4c). Samples CI-T-11, CI-T-12, CI-TE-8 and CI-TE-9 display microcracks in the matrix (Figure 4h). Sample CI-M-3 is characterised by fewer sandstone fragments and a more homogeneous texture compared with the rest of the samples.

The petrographic characteristics confirm the mineralogical and chemical composition of mortars. Samples with a large number of sandstone fragments correspond to the samples with the highest silica content. The matrix-supported texture of samples and the presence of fossils and calcareous rock fragments explain the high Ca content and the calcite identified by XRF and XRD, respectively. Sample CI-M-3 shows the lowest siliceous aggregate content as well as the highest presence of calcite as reflected in the Ca and LOI content obtained by chemical analysis (Table 2). The iron contents are associated with hematite and goethite present in sandstones and with the phyllosilicates in the matrix. The iron phases were not detected by XRD in most samples since the content is under the detection limit of the analytical method.

### 4.2. Pore System and Hydric Behaviour

Mercury intrusion porosimetry (MIP) values are summarised in Table 4. Samples CI-T-10 and CI-TE-9 show the highest porosity values (50.27% and 42.76%, respectively). Samples CI-T-11 and CI-TE-8 have the lowest PMIP values (20.44% and 2.48%, respectively) due to the presence of coarse aggregate with low porosity, resulting in an anomalous MIP value. The small sample size required in the measurement could include coarse and low porous aggregates affecting the results.

**Figure 5:**
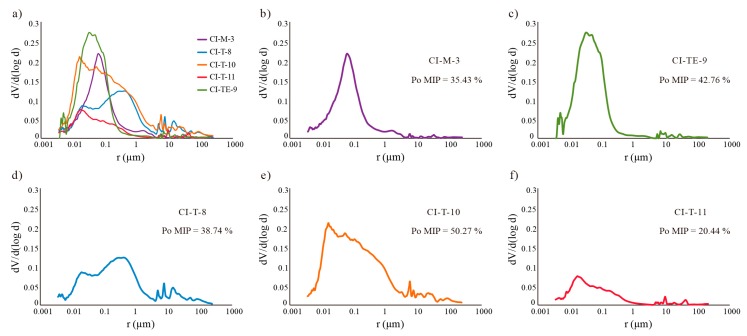
Mercury intrusion porosimetry pore size distribution curves for Irulegi Castle lime mortars: (**a**) all samples, (**b**) CI-M-3, (**c**) CI-TE-9, (**d**) CI-T-8, (**e**) CI-T-10, (**f**) CI-T-11. Pore radius (in µm) is represented versus incremental pore volume (in cm^3^/g).

Another fundamental characteristic of the pore system is the pore size distribution since the pore size affects the water circulation in the material [42,43]. MIP results indicate a large volume of small pores (0.01 < r < 1 μm) connected to larger pores (1 < r < 10 μm) in all samples (Figure 5). In this type of network, larger pores empty first whereas smaller pores remain full of liquid and dry more slowly [44]; therefore, the presence of two main families of pores strongly influences the drying behaviour of the material. However, pore size distribution is not the same in all samples (Figure 5a). Samples CI-M-3 and CI-TE-9 possess a nearly unimodal size distribution ranging between 0.01 µm and 1 µm although a second smaller family of pores ranging between 1 µm and 10 µm can be observed (Figure 5b,c). Sample CT-T-8 has a bimodal distribution of large pores of between 0.1 and 1 µm and a second smaller family between 0.01µm and 0.1 µm in size (Figure 5d). Samples CI-T-10 and CI-T-11 have a more heterogeneous pore size distribution but the Sample CI-T-10 possesses a large pore volume (Figure 5e,f).

**Figure 6:**
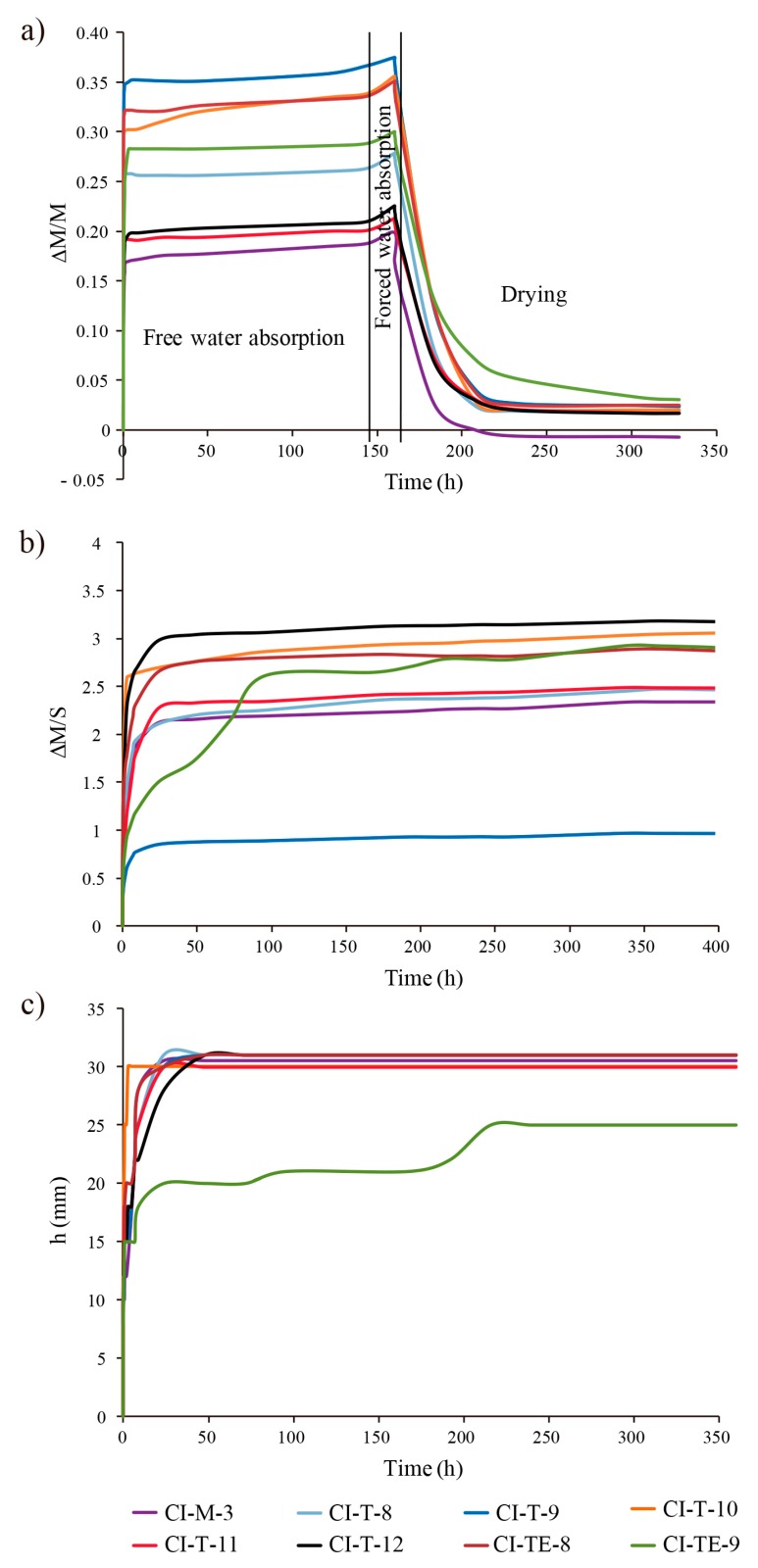
Hydric behaviours of Irulegi Castle lime mortars. (**a**) Free water absorption, forced water absorption and drying curves. Weight variation (ΔM/M) versus time (in hours). (**b**) Capillary uptake curves: Weight variation (ΔM/S) versus time (in hours) and (**c**) capillarity front curves. Height (in mm) versus time (in hours).

To complete the study on the pore system, hydric tests were also carried out (Figure 6). Table 4 shows the results of the hydric behaviour of mortar samples and their porosity accessible to water and mercury. Although the open porosity values obtained from the hydric tests (P_HT_) and by mercury intrusion porosimetry (P_MIP_) are very similar, the MIP values are always slightly higher. These differences are due to the fact that different liquids are used (H_2_O and Hg, respectively) and at different pressures (atmospheric pressure in the hydric tests and an injection pressure of 414 MPa in MIP analysis). Open porosity (P_HT_) has a direct effect on the durability of mortars because it allows water circulation within the structure favouring the entry of aggressive agents contained in the water. Therefore, the accessible porosity values are an important parameter to evaluate the possible deterioration of materials [45]. Samples CI-T-9, CI-T-10, CI-TE-8 and CI-TE-9 present the highest open porosity values (P_HT_ > 40%) while CI-M-3 shows the lowest values (35.43%).

Samples CI-T-9, CI-T-10, CI-TE-8 and CI-TE-9 display the highest free water absorption (A_b_, >28%) and forced water absorption (A_f_, >30%) levels whereas sample CI-M-3 shows the lowest values (A_b_ = 18.80% and A_f_ = 19.90%). Samples CI-T-8, CI-T-11 and CI-T-12 have intermediate water absorption and forced water absorption values (Table 4, Figure 6a). The lowest degree of pore interconnection values (A_x_) are in samples CI-T-10, CI-TE-8 and CI-TE-9 and, above all, in CI-T-9, indicating that water flows more easily inside mortars due to a better interconnection between the pores. On the contrary, CI-T-12 has the highest A_x_ values (almost 7%) indicating a more tortuous pore system hindering the flow of water inside the mortar (Table 4). The saturation coefficient (S) is directly related to the interconnectivity of the pores. In fact, the higher values are in samples CI-T-9, CI-TE-8 and CI-TE-9 (>93%). The absorption coefficient (C_a_) values are the highest (>15 g/min^0.5^) in samples CI-T-9, CI-T-10 and CI-TE-8. This value together with the degree of interconnection affects the water content capacity.

During the drying, two different drying phases occur. In the first drying phase, the evaporation of water from the wet surface is constant (constant rate drying) and the porosity has no significant influence on the drying rate [44,46]. In the second phase, drying depends on the pore radii and the degree of interconnection. When drying goes from constant velocity to a decreasing velocity the critical moisture content is reached and the drying rate changes. Then the water loss depends on the movement of water towards the surface through the capillary pores (falling rate period) [44,47]. Drying velocity is given by the desorption index (D_i_). Samples CI-T-9, CI-T-10 and CI-TE-8 present lower D_i_ values indicating fast drying, while CI-T-11, CI-T-12 and CI-TE-9 present with higher values took longer to dry (Table 4). Samples showing lower D_i_ values correspond to samples with lower degree of pore interconnection (A_x_) indicating that water flows easily outwards. On the contrary samples with high D_i_ and A_x_ values dry more slowly, indicating that water is retained longer in the mortar pore system, thus affecting its durability.

Regarding capillary uptake curves, samples absorb water quickly at the beginning of the test and as samples become saturated in water the velocity of capillary rise decreases and stabilizes reaching an equilibrium value (Figure 6b), following the most common capillary rise trend found for lime mortars [5]. The nonlineal curves showing two sections with different slopes are due to the presence of two main families of pores in the mortars [48] as MIP analysis revealed (0.01 μm < r < 1 μm and 1 μm < r < 10 μm). Samples CI-T-10 and CI-TE-8 have high capillarity coefficient (C_C_) values because they absorb water faster than samples CI-M-3 and CI-TE-9, which indeed show lower C_C_ values (Table 4). The capillary front reached the top of all samples after 48 h except in Sample CI-TE-9 where water did not reach the top until 216 h (nine days) after the beginning of the test (Figure 6c). However, this visual saturation does not coincide in time with the real saturation that occurs after 400 h (~16 days) (Figure 6b,c). This delay confirms the presence of two families of pores, which are filled at different velocities by water (smaller pores are filled first). Saturation is achieved when all connected pores are filled.

Samples CI-M-3 and CI-T-11 show the highest values of both bulk (ρ_Hb_) and skeletal (ρ_Hsk_) density (Table 4). Differences between skeletal and bulk densities are related to the porosity of mortars. This difference is greater in the more porous samples (CI-T-9, CI-T-10, CI-TE-8 and CI-TE-9).

### 4.3. Nondestructive Tests

Table 5 summarizes the ultrasound measurements in mortar samples. Considering lime mortar as an ideal two-phase media of aggregate embedded in a calcitic matrix, V_p_ values depend on the wave velocity both in the matrix and the aggregates, since ultrasonic wave propagation is different in each phase. The V_p_ decreases considerably when the wave propagates from an aggregate to the matrix [49]. No relationship between the V_p_ and aggregate size has not been found probably because aggregates act as a homogeneous structure considering size a constant parameter [50]. However, aggregate mineralogy is important in ultrasonic wave propagation velocity [51]. In fact, waves propagate more quickly through calcite (approximately 6660 m/s) than through quartz (approximately 5800 m/s) [52]. Therefore, P-wave velocity is directly related to the petrographic characteristic of the mortar. V_p_ values decrease in all mortar samples due to the presence of a large amount of siliceous aggregates, except in Sample CI-M-3, which contains the lowest amount of aggregates. Porosity also affects the ultrasonic wave propagation velocity since when the wave propagates from a solid (aggregates or matrix) through a gaseous medium (pores) the ultrasonic wave propagation velocity decreases [15]. Ultrasound data, indeed, are in agreement with the above-mentioned porosity results (Table 4 and Table 5). The presence of small cracks also affects V_p_, causing a fall in velocity [53], as in samples CI-T-11, CI-T-12, CI-TE-8 and CI-TE-9.

The total anisotropy coefficient of P-waves (ΔM_p_) confirms the textural homogeneity of CI-M-3 sample and the textural heterogeneity of the rest of the samples. Sample CI-M-3 gave the lowest ΔM_p_ value and sample CI-T-9 the highest (Table 5).

The chromatic parameters of each mortar are summarised in Table 6. Colorimetric analyses showed that the lightness value (L*) was lower for samples CI-M-3, CI-T-9, CI-T-10 and CI-T-11, while samples CI-T-12, CI-TE-8 and CI-TE-9 show the higher L* values. The chromatic axes (a* and b*) values tend towards to the light grey field due to a luminosity value close to 80. The a* values are very similar in all samples except for Sample CI-M-3 which presents the highest values. Samples CI-T-12 and CI-TE-9 have the lowest b* values. Lower L* is due to the presence of aggregates, phyllosilicate phases and iron oxides, as petrographic and XRD analyses indicate.

According to the measured parameters, samples CI-T-9, CI-T-10, CI-TE-8 and CI-TE-9 (South-Southeast face of the Tower Keep) present the highest free water absorption values (A_b_ > 25%), high porosity values (P_HT_ and P_MIP_ > 40%) with good pore interconnection (lowest A_x_ values), favouring the incorporation of dissolved salts and gaseous species such as CO_2_ into the material that could deteriorate the mortar. Samples CI-T-11 and CI-T-12 (Southwest wall of the Tower Keep) display the worst pore interconnection (higher A_x_ values) and dry more slowly (higher D_i_ values), indicating a longer water retention also affecting mortar durability. Only sample CI-M-3 from the West perimeter wall shows less potential of deterioration due to the low free water absorption values (A_b_ < 19%), fast drying and lower porosity (P_HT_ and P_MIP_ ~32%).

Additionally, environmental factors condition mortar durability due to the presence of water both as humidity (water vapour) and as rainfall (liquid water). The climatic conditions at Irulegi Castle favour mortar deterioration since the average annual precipitation is around 858 mm and in 10 months rainfall is above 50 mm. The annual average humidity is ~76% but in summer periods the humidity is higher than the average values. Moreover, the average temperature is 11.8 °C but the absolute average minimum and maximum temperatures are −12.4 °C and 39.9 °C, respectively [54]. The wide variation between absolute maximum and minimum temperature together with the high humidity favour the physical weathering of mortars. Material exposure to temperature variations leads to thermal expansion and temperatures below freezing result in frost wedging causing cracking of mortars. Additionally, the persistent rainfall favours dissolved salts and CO_2_ incorporation into the mortars producing crystal growth and carbonate species dissolution, respectively.

## 5. Conclusions and Perspectives

The mineralogical, chemical and physical properties of eight archaeological lime mortar samples from Irulegi Castle were determined to enable an understanding of their susceptibility to deterioration.

High pore volume in the 0.01 to 1µm size range is one of the reasons for the durability problems that the studied mortars might suffer in the future, since smaller pores retain water longer and dry more slowly. Related with their pore system, samples from the Tower Keep show high susceptibility to deterioration compared with the West perimeter wall sample. Samples from the south-southeast face of the Tower Keep show higher water absorption capacity and porosity (with good pore interconnection), while samples from the Southwest wall present the worst pore interconnection and dry more slowly.

Not only do the intrinsic features of the original mortars of Irulegi Castle, but also the local environmental exposure conditions, affect mortar deterioration. The persistent rainfall during the year, high humidity and temperature variations in this area certainly contribute to weathering processes in the original mortars. To design durable and compatible repair material for this castle, original mortar characteristics and environmental conditions should be taken into account.

Considering that the studied mortars are archaeological medieval mortars, differences in the physical properties among nearby samples would not necessarily indicate different mortar manufacturing processes but could instead correspond to the typical heterogeneity of this type of material.

This study was able to determine the chemical-mineralogical characteristics and physical properties of the original historical mortars at Irulegi Castle, with positive implications for the design of compatible and durable repair mortar in future interventions. It will be essential to select the most appropriate mortar composition to ensure a satisfactory and long-lasting repair intervention. Compatibility, durability, authenticity and reversibility of the repair materials are indeed crucial requirements in any restoration work to be carried out in the future in this castle.

In the future, additional decay tests, including salt crystallization, wet and dry cycles, rainfall exposure and freeze-thaw cycles, should be carried out to assess the deterioration processes due to environmental agents in the area.

## Figures and Tables

**Table 1:** Studied samples from different structures at Irulegi Castle.

Archaeological Structure	Sample
West perimeter wall	CI-M-3
Tower Keep	CI-T-8
	CI-T-9
	CI-T-10
	CI-T-11
	CI-T-12
Tower Keep extension	CI-TE-8
	CI-TE-9

**Table 2:** Results of mineralogical composition of bulk mortar determined by X-ray diffraction. Cal: calcite, Qtz: quartz, Fsp: feldspar s.l.; Phy: phyllosilicates s.l.; Fe ox: iron oxides. The amount was expressed as follows: ****: predominant compounds; ***: high proportion; **: medium proportion; *: low proportion; tr: trace; -: undetected.

Sample	Cal	Qtz	Phy	Fsp	Hem
CI-M-3	****	**	*	tr	-
CI-T-8	***	**	*	tr	-
CI-T-9	**	****	*	*	tr
CI-T-10	***	**	*	tr	-
CI-T-11	***	**	*	*	-
CI-T-12	***	**	*	tr	-
CI-TE-8	***	**	*	tr	-
CI-TE-9	***	**	*	*	*

**Table 3:** Result of chemical composition of major elements of bulk mortar determined by X-ray fluorescence expressed as oxides in wt %. The iron content has been expressed as total Fe_2_O_3_t. LOI: loss on ignition (%).

	SiO_2_	Al_2_O_3_	Fe_2_O_3_t	MnO	MgO	CaO	Na_2_O	K_2_O	TiO_2_	P_2_O_5_	SO_3_	LOI
CI-M-3	31.95	3.27	1.82	0.06	0.63	31.69	0.08	0.56	0.28	0.06	0.11	28.86
CI-T-8	38.08	2.95	1.25	0.03	0.60	28.77	0.09	0.74	0.23	0.12	0.06	26.57
CI-T-9	40.60	3.26	1.34	0.03	0.69	26.90	0.07	0.76	0.25	0.13	0.07	24.40
CI-T-10	38.67	3.42	1.39	0.03	0.74	26.93	0.11	0.79	0.25	0.10	0.03	25.07
CI-T-11	36.07	3.42	1.52	0.03	0.70	26.49	0.08	0.76	0.25	0.27	0.07	26.39
CI-T-12	38.44	2.55	1.37	0.03	0.55	31.03	0.08	0.57	0.2	0.09	0.07	23.99
CI-TE-8	35.46	2.32	1.16	0.03	0.65	29.39	0.04	0.43	0.18	0.08	0.10	25.98
CI-TE-9	37.40	2.37	1.29	0.03	0.56	32.29	0.06	0.47	0.2	0.08	0.05	23.97

**Table 4:** Parameters obtained by hydric tests (HT) and mercury intrusion porosimetry (MIP) of Irulegi Castle lime mortars. A_b_: Free water absorption (%); A_f_: forced water absorption (%); A_x_: degree of pore interconnection (%); S: Saturation coefficient (%); C_a_: absorption coefficient (g/min^0.5^); D_i_: drying index; P_HT_ and P_MIP_: open porosity (%); ρ_Hb_: bulk density (g/cm^3^); ρ_Hsk_: skeletal density (g/cm^3^); C_c_: capillarity coefficient (g/cm^2^ min^0.5^); H_c_: Height of the water level during capillary uptake (mm s^−0.5^).

	CI-M-3	CI-T-8	CI-T-9	CI-T-10	CI-T-11	CI-T-12	CI-TE-8	CI-TE-9
A_b_	18.80	26.35	36.63	33.83	20.08	20.94	33.61	28.86
A_f_	19.90	27.87	37.45	35.57	21.29	22.50	35.11	30.04
A_x_	5.54	5.43	2.20	4.91	5.68	6.95	4.28	3.92
S	89.17	92.00	93.68	90.17	91.08	90.11	93.11	94.26
C_a_	9.40	13.18	18.31	16.91	10.04	10.47	16.81	14.43
D_i_	0.264	0.266	0.261	0.262	0.270	0.269	0.262	0.267
ρ_Hb_	1.62	1.39	1.24	1.27	1.62	1.51	1.31	1.39
ρ_Hsk_	2.39	2.26	2.33	2.31	2.47	2.28	2.41	2.38
C_c_	0.010	0.013	0.009	0.021	0.011	0.018	0.021	0.008
H_c_	0.42	0.52	0.52	0.77	0.65	0.65	0.65	0.52
P_HT_	32.25	38.63	46.55	45.06	34.48	33.88	45.86	41.68
P_MIP_	35.43	38.74	-	50.27	20.44	-	2.48	42.76

**Table 5:** Results of ultrasonic wave propagation through lime mortars from Irulegi Castle. V_P1_, V_P2_ and V_P3_ (in m/s): P-wave velocity in three orthogonal directions; ΔM_p_: total anisotropy coefficient of P-waves.

	CI-M-3	CI-T-8	CI-T-9	CI-T-10	CI-T-11	CI-T-12	CI-TE-8	CI-TE-9
V_P1_	1815.48	186.75	178.77	130.80	158.19	150.49	178.77	151.83
V_P2_	1828.91	197.60	340.66	134.20	159.79	169.49	191.62	183.43
V_P3_	1820.90	197.60	358.70	132.78	197.37	157.36	203.82	177.51
ΔM	0.52	5.49	48.88	2.01	11.43	7.92	9.58	15.87

**Table 6:** Chromatic parameters of mortar samples. Lightness (L*), chromatic coordinates (a* and b*), chroma (C*), hue angle (H).

	CI-M-3	CI-T-8	CI-T-9	CI-T-10	CI-T-11	CI-T-12	CI-TE-8	CI-TE-9
L*	72.31	78.31	76.76	73.69	73.41	80.79	80.55	80.24
a*	3.22	2.36	2.35	2.95	2.38	2.15	2.81	2.04
b*	13.79	11.83	11.26	14.67	12.8	9.63	12.86	9.76
C*	14.16	12.07	11.51	14.96	13.02	9.86	13.17	9.97
H	76.85	78.71	78.06	78.63	79.46	77.45	77.82	78.22
